# Three-Dimensional Regeneration of Patient-Derived Intestinal Organoid Epithelium in a Physiodynamic Mucosal Interface-on-a-Chip

**DOI:** 10.3390/mi11070663

**Published:** 2020-07-07

**Authors:** Yong Cheol Shin, Woojung Shin, Domin Koh, Alexander Wu, Yoko M. Ambrosini, Soyoun Min, S. Gail Eckhardt, R. Y. Declan Fleming, Seung Kim, Sowon Park, Hong Koh, Tae Kyung Yoo, Hyun Jung Kim

**Affiliations:** 1 Department of Biomedical Engineering, The University of Texas at Austin, Austin, TX 78712, USA; yongcheol.shin@austin.utexas.edu (Y.C.S.); wjshin@utmail.utexas.edu (W.S.); dominkoh@utexas.edu (D.K.); alex.wu.2@utmail.utexas.edu (A.W.); yokoambrosini@utmail.utexas.edu (Y.M.A.); soyoun.min@austin.utexas.edu (S.M.); 2 Department of Oncology, Dell Medical School, The University of Texas at Austin, Austin, TX 78712, USA; gail.eckhardt@austin.utexas.edu (S.G.E.); declan.fleming@austin.utexas.edu (R.Y.D.F.); 3 Department of Surgery and Perioperative Care, Dell Medical School, The University of Texas at Austin, Austin, TX 78712, USA; 4 Severance Fecal Microbiota Transplantation Center, Severance Hospital, Department of Pediatrics, Yonsei University College of Medicine, Seoul 03722, Korea; pedks@yuhs.ac (S.K.); sowon81@yuhs.ac (S.P.); khong@yuhs.ac (H.K.); 5 Department of Computer Art, College of Art and Technology, Chung-Ang University, Seoul 06974, Korea; yootaekyung@cau.ac.kr; 6 Department of Medical Engineering, College of Medicine, Yonsei University, Seoul 03722, Korea

**Keywords:** gut-on-a-chip, organoid, mucosal interface, physiodynamic, multiaxial deformation, microbiome, co-culture, disease modeling

## Abstract

The regeneration of the mucosal interface of the human intestine is critical in the host–gut microbiome crosstalk associated with gastrointestinal diseases. The biopsy-derived intestinal organoids provide genetic information of patients with physiological cytodifferentiation. However, the enclosed lumen and static culture condition substantially limit the utility of patient-derived organoids for microbiome-associated disease modeling. Here, we report a patient-specific three-dimensional (3D) physiodynamic mucosal interface-on-a-chip (PMI Chip) that provides a microphysiological intestinal milieu under defined biomechanics. The real-time imaging and computational simulation of the PMI Chip verified the recapitulation of non-linear luminal and microvascular flow that simulates the hydrodynamics in a living human gut. The multiaxial deformations in a convoluted microchannel not only induced dynamic cell strains but also enhanced particle mixing in the lumen microchannel. Under this physiodynamic condition, an organoid-derived epithelium obtained from the patients diagnosed with Crohn’s disease, ulcerative colitis, or colorectal cancer independently formed 3D epithelial layers with disease-specific differentiations. Moreover, co-culture with the human fecal microbiome in an anoxic–oxic interface resulted in the formation of stochastic microcolonies without a loss of epithelial barrier function. We envision that the patient-specific PMI Chip that conveys genetic, epigenetic, and environmental factors of individual patients will potentially demonstrate the pathophysiological dynamics and complex host–microbiome crosstalk to target a patient-specific disease modeling.

## 1. Introduction

The gastrointestinal (GI) epithelial barrier is a physical interface that undergoes constant exposures to the exogenous molecules and antigens (i.e., pathogens, nutrients, or toxins). Across the intestinal mucosal interface, complex host–microbiome crosstalk, immune responses, and pharmacological interactions occur to maintain intestinal homeostasis [1]. Hence, the recreation of an intestinal mucosal interface in vitro is important to demonstrate pathophysiological responses at various GI diseases to predict the immunomodulatory effect of the gut microbiome [2] or validate drug effectiveness [3].

There have been a number of in vitro human intestine models [4,5,6,7,8,9], where the human gut-on-a-chip has shown innovative features to simulate physiological biomechanics [10], three-dimensional (3D) epithelial morphogenesis [11,12], host–microbiome ecosystem [10,13], anoxic–oxic interface (AOI) [14], epithelial barrier function, and inflammatory immune responses [15,16]. The implementability of the gut-on-a-chip has been improved by integrating human intestinal organoids that reflect the patient’s genetic heritage [17,18], disease pathophysiology [19], and host–gut microbiome interactions [20]. This integrative strategy is particularly important for disease modeling because the recreation of mucosal tissue interface using patient’s samples allows us to deploy individual disease factors (e.g., dysbiotic gut microbiome, activated immune cells) in a defined space and time. However, it has been challenging to standardize and disseminate the microphysiological system (MPS)-based disease modeling, because the culture protocol and device manufacturing are technically nascent [21,22]. Furthermore, advanced features such as in vivo-like hydrodynamic flow [23,24,25], multiaxial mechanical deformations [26,27], controlled oxygen gradient in a mucosal interface [28], and robust co-culture with anaerobic gut bacteria [29] are essentially required.

In this study, we report a 3D physiodynamic mucosal interface-on-a-chip (PMI Chip) that provides a technical foundation to build the patient-specific models of major human chronic GI disease such as inflammatory bowel disease (IBD), including Crohn’s disease (CD), ulcerative colitis (UC), and colorectal cancer (CRC). Using a modular PMI Chip, we verified the robust 3D regeneration of intestinal epithelium using biopsy-derived organoids from CD, UC, and CRC patients as well as normal individuals. We also characterized the physiological movement and hydrodynamic patterns by using real-time imaging and computational simulation. Finally, we demonstrated the co-culture of the human fecal microbiome in a patient-specific mucosal interface, which will be critical to recapitulate disease-associated host–microbiome crosstalk as a proof of principle. We envision that the PMI Chip may enable developing an advanced in vitro model that demonstrates the signature of initiation, progression, and development of CD, UC, and CRC at various disease milieus.

## 2. Materials and Methods

### 2.1. Microfabrication of a Device

A polydimethylsiloxane (PDMS) mixture (silicone elastomer:curing agent = 15:1 (*w*/*w*); Sylgard 184, Dow Corning, Midland, MI, USA) was poured on the molds for creating an upper layer, a porous membrane, and a lower layer [10,13,30,31]. Then, the cured PDMS pieces of each compartment baked at 60 °C for 6 h were demolded and punched (2 mm in diameter; Harris Uni-Core, GE Healthcare, Chicago, IL, USA). To assemble an upper layer, a porous membrane, and a lower layer, each compartment was sequentially treated with a corona treater (BD-20A; Electro-Technic Products, Chicago, IL, USA) or a plasma cleaner (COVANCE-1MPR; Femto Science Inc., Gyeonggi, Korea) and aligned under a stereoscope (M50, Leica, Wetzlar, Germany) as previously demonstrated [10,13,30,31,32]. After the assembly, the inlets and outlets of a device are connected to silicone tubing (Tygon 3350, ID 1/32”, OD 3/32”, Saint-Gobain, Courbevoie, France) using a bent metal connector (18-gauge blunt-end needle, Shintop; 90-degree angled). Each port is sealed with epoxy glue (Devcon, Hartford, CT, USA) to avoid unnecessary leakage.

### 2.2. Computational Simulation

A 3D model of a convoluted PMI Chip was created by AutoCAD (Autodesk, San Rafael, CA, USA) and exported into COMSOL Multiphysics 5.4 (COMSOL Inc., Stockholm, Sweden) to estimate fluid hydrodynamics in terms of flow velocity and fluid shear stress. The upper and lower layers were completely separated by placing 20 µm of a thin layer, assuming that the effect of the pores on the membrane is negligible. The microfluidic module of COMSOL was used for the simulation. The laminar flow physics was selected without the effects of gravity governed by the Navier–Stokes equation to simulate the flow within a PMI chip. The fluid in both upper and lower microchannels was set to water. The wall of a microchannel was set to the fixed wall without deformation (no-slip boundary condition). The volumetric flow rate at the upper and lower layers was set to 50 μL/h, and the flow velocity and shear stress were calculated using COMSOL and Equation (1), respectively [10]:

(1) $$\mathrm{Shear}\;\mathrm{stress}\;(\mathrm{dyne}/{\mathrm{cm}}^{2})=\frac{6µQ}{h^{2}w}$$

where µ is the viscosity of the culture medium (g/cm·s), *Q* is the volumetric flow rate (cm^3^/s), and *h* (cm) and *w* (cm) are the height and width of the microchannel, respectively. For data presentation, time-independent data at a stationary phase were calculated and visualized. The fluid residence time in the microchannel was calculated by dividing the volume of the upper microchannel (8.93 mm^3^ for PMI Chip, 5 mm^3^ for gut-on-a-chip) by the flow rate (30–100 μL/h). A pressure difference between the upper and lower microchannels was calculated by subtracting the pressure values (P) of each microchannel obtained using Equation (2) [33]:

(2) $$P\;(\mathrm{Pa})=\frac{12µQL}{WH^{3}}{\left[1-\frac{192H}{\pi^{5}W}\tanh\left(\frac{\pi W}{2H}\right)\right]}^{-1}$$

### 2.3. Organoid Culture

Human intestinal organoids or tissue samples were obtained to establish organoid lines from various institutions as follows; normal organoid (C103) from Baylor College of Medicine, ulcerative colitis (UC) organoid (UC003; IRB-4-2017-0223) from Yonsei University College of Medicine, Crohn’s disease (CD) organoid (CD7517) from The University of Chicago, and colorectal cancer (CRC) organoid (CRC001; IRB-2017-06-0114) from the Dell Medical School at The University of Texas at Austin. All samples were obtained with informed consent from the donors under the approved documents from the individual Institutional Review Board (IRB) at each institution. The C103 and CD7517 were obtained as the established organoid lines, whereas the UC003 and CRC001 lines were established in the laboratory from obtained tissue samples. The organoids were derived from either endoscopic biopsies (normal, UC, and CD organoids) or surgically resected colon tissues (CRC organoid). The isolated organoids were embedded in 30 µL of Matrigel (Corning) and routinely maintained with 500 µL of the organoid culture medium in a 24-well plate (Corning) in a humidified incubator with 5% CO_2_ at 37 °C [34].

The basal medium was prepared in Advanced Dulbecco’s Modified Eagle Medium (DMEM)/F12 (Gibco) by supplementing 10 mM 4-(2-hydroxyethyl)-1-piperazineethanesulfonic acid (HEPES; Gibco), 1× GlutaMAX (Invitrogen), 100 units/mL penicillin (Thermo Fisher Scientific), and 100 μg/mL streptomycin (Thermo Fisher Scientific). Conditioned medium was prepared by culturing Wnt3a-producing L cells (L-Wnt3a; ATCC, CRL 2647), R-spondin1 (Rspo1) cells (Trevigen), and Noggin-secreting HEK293 cells (Baylor College of Medicine). To produce Wnt3a conditioned medium, L-Wnt3a cells were cultured in a T75 tissue culture flask (CellTreat Scientific Products) with 10 mL of Advanced DMEM/F12 containing 8% (*v*/*v*) fetal bovine serum (FBS; Gibco) and 1× GlutaMAX in a humidified CO_2_ incubator at 37 °C. After 4 days of incubation, the first batch of conditioned medium was harvested, centrifuged at 1000× *g* at 4 °C for 10 min, and filtered through a 0.22 µm polyvinylidene fluoride (PVDF) membrane filter (Millipore). To collect the second batch of conditioned medium, 10 mL of fresh medium was added into a T75 flask, and cells were incubated for an additional 3 days. The second batch of conditioned medium was collected, centrifuged, and filtered as described above. Then, the first and second batches of conditioned medium were mixed, and the mixture was aliquoted into conical tubes (Corning) for storage at −80 °C. The Rspo1 and Noggin conditioned media were prepared by culturing Rspo1 and Noggin-secreting HEK293 cells, respectively, in a T75 flask with 20 mL of Advanced DMEM/F12 containing 8% (*v*/*v*) FBS and 1× GlutaMAX in a humidified CO_2_ incubator at 37 °C for 7 days. Each medium was harvested, centrifuged at 1000× *g* at 4 °C for 10 min, filtered through a 0.22 µm PVDF membrane filter, and then stored at −80 °C. The organoid culture medium was prepared by mixing conditioned media of Wnt3A, Rspo1, and Noggin in the basal medium at a volume ratio of 75:10:5:10 (*v*/*v*). In this mixture, we supplemented murine recombinant epidermal growth factor (EGF) (50 ng/mL; Peprotech), SB202190 (30 μM; Sigma Aldrich), A-8301 (500 nM; Sigma Aldrich), Gastrin (10 nM; Sigma Aldrich), *N*-acetylcysteine (1 mM; MP Biomedicals), nicotinamide (10 mM; Sigma Aldrich), N2 (1×; Gibco), and B27 (1×; Gibco). The culture medium was changed every other day to organoids that are routinely cultured in a 24-well plate in a humidified CO_2_ incubator at 37 °C. For the passage, the organoids embedded in the Matrigel were incubated in 500 µL of Cell Recovery Solution (Corning) at 4 °C for 30 min, harvested in a sterile 15 mL conical tube, spun down (at 100× *g* at 4 °C for 5 min), and then collected after aspiration of the supernatant. To prepare organoid fragments, the organoid pellets were incubated with 1 mL of TrypLE Express solution (Gibco) at 37 °C for 2 min in a water bath. After neutralization by adding 10 mL of Advanced DMEM/F12 containing 10% (*v*/*v*) FBS, organoids were mechanically fragmented using a P1000 pipette. The fragmented organoids were centrifuged at 100× *g* at 4 °C for 5 min and resuspended with Matrigel on ice; then, 30 µL of the organoid suspension was dropped into a well of a 24-well plate, incubated for gelation at 37 °C for 10 min, and then submerged with 500 µL of the organoid culture medium for growth.

### 2.4. Microfluidic Cultures in a PMI Chip

Prior to seeding the Caco-2 and organoid-derived epithelial cells in a PMI device, the surface of microchannels was activated and coated with extracellular matrix (ECM) proteins. Briefly, microchannels were sterilized with 70% (*v*/*v*) ethanol and dried at 60 °C overnight. The device was then exposed to UV/ozone (Jelight Company Inc.) for 1 h, treated with 1% (*v*/*v*) polyethyleneimine (PEI; Sigma Aldrich) solution for 10 min at room temperature followed by 0.1% (*v*/*v*) glutaraldehyde (GA; Electron Microscopic Sciences) solution for 20 min at room temperature, washed by flowing deionized water using a disposable syringe through the tubing, and dried at 60 °C overnight. After cooling down the device setup, microchannels were filled with an ECM solution containing 1% (*v*/*v*) Matrigel and 30 µg/mL of collagen I (Fisher Scientific), incubated at 37 °C in a humidified CO_2_ incubator for 2 h, rinsed out with basal medium at 100 μL/h for 1 h using a syringe pump, and then used for seeding the Caco-2 and dissociated organoid cells.

Human intestinal epithelial Caco-2 cells (Harvard Digestive Disease Center) were routinely cultured in Dulbecco’s Modified Eagle Medium (DMEM; Gibco) supplemented with 20% (*v*/*v*) FBS and antibiotics (100 U/mL penicillin and 100 μg/mL streptomycin; Gibco). To culture Caco-2 cells in the PMI Chip, cells harvested from a T75 tissue culture flask were seeded into the upper microchannel (final cell density, approximately 1 × 10^6^ cells/mL) and incubated in a humidified CO_2_ incubator at 37 °C without flow for 1 h. Then, the attached cells were cultured under constant flow (50 μL/h) and cyclic mechanical strain. The cyclic mechanical strain was applied using a Flexcell FX-5000 tension system (Flexcell International Corporation) with 5% of cell strain at a frequency of 0.15 Hz [10,30]. The percentage of cell strain indicates the average elongation (%) of the microchannel, which corresponds to the cell deformation [10].

To culture intestinal organoids in the PMI Chip, organoids fully grown for 7 days were dissociated using the method identical to the passage. Dissociated organoid cells were filtered through a 300-µm cell strainer (pluriSelect), centrifuged at 100× *g* at 4 °C for 5 min, resuspended in the organoid culture medium (final cell density, approximately 1 × 10^7^ cells/mL), and then seeded into the ECM-coated upper microchannel of the device. To form an organoid-derived monolayer, the device setup was incubated in a humidified CO_2_ incubator at 37 °C for overnight; then, it was flowed with the organoid culture medium to the upper microchannel at 50 μL/h for approximately 2 days until the cells create a full intact monolayer. To induce 3D morphogenesis, the organoid culture medium was perfused to both upper and lower microchannels at 50 μL/h, and the cyclic mechanical strain (5% in cell strain, 0.15 Hz in frequency) was applied.

### 2.5. Analysis of Multiaxial Stretching Motion

To visualize the multiaxial stretching motions in the PMI Chip, phase contrast images of the microchannel were acquired using an inverted light microscope (DMi1, Leica). Next, we set out dots (25–30 random locations) to trace the elongated positions before (i.e., 0% strain; red) and after stretching motions (5% cell strain; light blue) using a module of particle tracking in ImageJ (Ver. 1.52p). To quantify the elongation intensity of the microchannel at each curved corner, 3 guidelines were drawn across the microchannel at different angles. The percentage of elongation was calculated at three different locations in each line based on the following Equation (3):

(3) $$\mathrm{Elongation}\;(\%)=\frac{L_{elongated}-L_{0}}{L_{0}}\times 100$$

where *L*_elongated_ and *L*_0_ are the lengths at 5% and 0% strain, respectively. To quantitatively analyze the stretching directions of a PMI Chip and a gut-on-a-chip, we applied a grid with 16 squared compartments to divide the individual phase contrast images taken in both non-stretched (No Str) and stretched conditions (+Str) in a PMI Chip and a gut-on-a-chip, respectively. Next, we randomly positioned two dots in each compartment to track the directionality of stretching by applying a straight line between the dots to measure a stretching angle between the horizontal axis of the vacuum-driven elongation and the direction of stretched deformation. The frequency (%) was calculated as the ratio between the number of dots in each angle range (10-degree steps) and the total number of dots counted on the grid.

### 2.6. Visualization of the Epithelial Elongation

An overlaid video that visualizes the extension of an epithelial layer was created by mapping the degree of elongation as a function of color density. The coding was performed by Expression in the Adobe After Effects. We used a cell stretching movie using an inverted phase contrast microscope (DMi1) equipped with a 5× objective (NA 0.12; Leica). An array of motion tracking points was designated uniformly at every 60 pixels onto the target area in the movie frame; then, each motion tracking point was analyzed. Next, we attached the radial gradient of red dots to each motion tracking point. Finally, the opacity of each red point was adjusted in response to the displacement of the motion tracking point. The degree of elongation is displayed as a function of red color intensity, where higher color intensity indicates longer elongation.

### 2.7. Verification of the Effect of Elongation on the Flow Pattern

To verify the effect of elongation on the flow pattern, we visualized fluid flow in a PMI Chip with or without stretching motions. A colloidal solution containing fluorescent beads (1000× dilution; Fluoro-Max Dyed Green Aqueous Fluorescent Particles; mean diameter, 1 μm; Thermo Fisher Scientific) was perfused to the upper microchannel in a PMI Chip at 50 μL/h using a syringe pump (BS-8000 infusion pump, Braintree Scientific). The lower microchannel was flowed with a bead-free culture medium. Next, we monitored the trajectory of fluorescent beads in the presence or absence of cyclic multiaxial deformations (5%, 0.15 Hz) by acquiring consecutive time-lapse images using a confocal microscope (DMi8, Leica) for 1 min at the time interval of 1 s. We analyzed the trajectory of fluorescence beads (>65 beads) using the Mosaic Particle Tracker plugin (ImageJ, Ver 1.52p). The directional movements of individual fluorescence beads were quantitatively profiled as a function of migration angle using the Directionality plugin (ImageJ, Ver 1.52p). Variance (σ^2^) was calculated as the square of the standard deviation (SD). Alternatively, we analyzed time-lapse phase contrast images to manually track the trajectory of floating cell debris in the upper convoluted microchannel.

### 2.8. Morphological Analysis

The morphology of organoid-derived or Caco-2 intestinal epithelium was observed and acquired using an inverted light microscope (DMi1) or a differential interference contrast (DIC) microscope (DMi8). For immunofluorescence microscopic analysis, cells cultured in a device were fixed with 4% (*w*/*v*) paraformaldehyde (Electron Microscopy Science) for 20 min, permeabilized with 0.3% (*v*/*v*) Triton X-100 (Sigma Aldrich) for 30 min, and blocked with 2% (*w*/*v*) bovine serum albumin (BSA, Sigma Aldrich) solution at room temperature for 2 h. Next, cells were sequentially incubated with the primary (at 4 °C, overnight) and secondary antibodies (at room temperature, 1 h, under light protected), followed by the counterstaining for visualizing both F-actin (Alexa Fluor 647 phalloidin, 165 nM; Thermo Fisher Scientific) and nuclei (4′,6-diamidino-2-phenylindole (DAPI) at 1 µg/mL; Fisher Scientific). Primary antibodies were used to stain MUC2 (mouse anti-MUC2 monoclonal antibody, 2 µg/mL; Santa Cruz Biotechnology), Ki-67 (rabbit anti-Ki-67 polyclonal antibody, 1 µg/mL; Abcam), carcinoembryonic antigen (CEA) (mouse anti-CEA monoclonal antibody, 100× dilution; Abcam), and CD133 (rabbit anti-CD133 antibody, 2 µg/mL; Abcam). After washing microchannels with phosphate-buffered saline (PBS, Ca^2+^- and Mg^2+^-free; Gibco), secondary antibodies (DyLight 488-conjugated goat polyclonal anti-mouse IgG, 2.5 µg/mL; Alexa Fluor 555-conjugated goat polyclonal anti-rabbit IgG, 2.5 µg/mL; Abcam) were applied. To highlight E-cadherin, Alexa Fluor 488-conjugated monoclonal anti-E-Cadherin antibody (100× dilution; BD Biosciences) was used. The stained cells were imaged using a laser-scanning confocal microscope (DMi8) equipped with a 25× objective (water emulsion, NA 0.95; Leica). All the acquired images were analyzed and modified into 3D angled views using LAS X software (Leica). To measure the height of 3D epithelium, vertical crosscuts of z-stacked (z-axis scanning) images were obtained, and the linear length between the base and the apical surface of the epithelium was quantified.

### 2.9. Co-Culture of Fecal Microbiome

A fecal specimen was obtained from a healthy donor (Yonsei University College of Medicine, IRB-4-2017-0223). The frozen fecal specimen was transferred into an anaerobic glove box (Bactron 300; Sheldon Manufacturing) maintained under 90% N_2_, 5% CO_2_ and 5% H_2_ of the gas mixture, allowed to thaw on ice for 3 min, then homogenized with 0.9% (*w*/*v*) saline containing glycerol (final concentration, 12.5% *v*/*v*) and L-cysteine (final concentration, 0.1%, *w*/*v*) using a homogenizer (Bel-Art) for 2 min. The fecal homogenate was filtered (sequential cut-off at 500 and 300 µm; pluriSelect) to remove fecal debris; then, it was immediately frozen at –80 °C until use. The bacterial cell density in a crude fecal suspension was determined using a LIVE/DEAD BacLight assay kit (Molecular Probes) under confocal microscopy.

When Caco-2 intestinal epithelium was fully grown with the 3D structure in a PMI Chip, both the upper and lower microchannels were preconditioned with antibiotic-free culture medium for 12 h. Before the bacterial seeding, the anoxic culture medium containing L-cysteine (final concentration at 0.1%, *w*/*v*) was introduced to the upper microchannel to establish the AOI [14]. The processed fecal microbial suspension was diluted in an anoxic culture medium to make a final cell density of 5 × 10^4^ cells/mL. Then, the fecal microbiome was seeded into the upper microchannel of the AOI-preconditioned PMI Chip, and a whole setup was incubated in a humidified CO_2_ incubator at 37 °C without perfusion for 1 h. Then, the culture medium was resumed to flow (100 µL/h) to both upper and lower microchannels under cyclic mechanical strains (5%, 0.15 Hz). To assess the mucosal barrier function, the transepithelial electrical resistance (TEER) was measured using Ag/AgCl electrodes (A-M Systems) connected to an ohmmeter (87V Industrial Multimeter; Fluke Corporation) intermittently. The TEER values were normalized using Equation (4) [16]:

(4) $$\mathrm{Normalized}\;\mathrm{TEER}=\frac{\left(\Omega_{t}-\Omega_{blank}\right)}{\left(\Omega_{0}-\Omega_{blank}\right)}$$

where *Ω_t_* is the resistance value of a PMI Chip that contains the host and bacterial cells at each time point of *t*, *Ω_blank_* is the blank resistance value of a microfluidic device without any cells, and *Ω_0_* is the resistance at Day 0 (i.e., before seeding the microbial cells).

### 2.10. Statistical Analysis

All the quantitative data were expressed as the mean ± SD. A one-way analysis of variance (ANOVA) followed by a Bonferroni test was applied using OriginPro 8.0 software (Origin Laboratories). Differences between groups were considered statistically significant when *p* < 0.05.

## 3. Results

### 3.1. Design of a PMI Chip and its Fluid Dynamics

**Figure 1:**
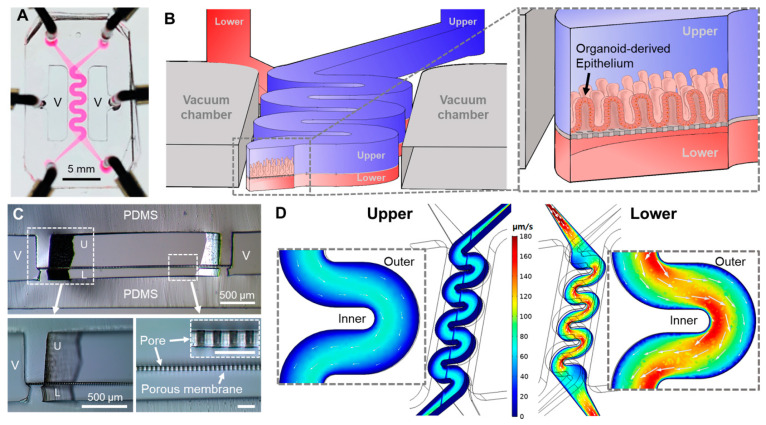
The configuration of a physiodynamic mucosal interface-on-a-chip (PMI Chip). (**A**) A photograph of a PMI Chip device. The culture medium was filled in the upper and lower microchannels to visualize the convoluted design of microchannels. (**B**) A 3D layout of a PMI Chip and a zoom-in cross-sectional view illustrate the structural configuration of the convoluted microchannel that contains an organoid-derived 3D epithelial layer. The upper (blue) and lower (red) microchannels mimic the lumen and capillary compartments in the gut, respectively. (**C**) The cross-sectional micrographs of a PMI Chip (upper panel) and the zoom-in insets (lower panel) that highlight the white dashed boxes in the upper panel. An inset in the lower right panel shows a cross-cut view of a PDMS porous membrane (Bar, 50 μm). V, vacuum chamber; U, upper microchannel; L, lower microchannel. (**D**) The velocity profiles of fluid flow (unit, μm/s) in the upper and lower microchannels at 50 μL/h. White arrows in the zoom-in insets indicate the direction of the fluid flow and the magnitude of a linear flow rate.

We designed a new PDMS-based microfluidic device that has two superposed convoluted microchannels divided by a PDMS porous membrane to build a PMI Chip (Figure 1A). Under physiological motions and flow, we aimed to use this PMI Chip to better mimic the complex dynamics of intestinal biomechanics. We also targeted to recreate the physiodynamic mucosal interface in a “patient’s avatar chip” using an organoid-derived epithelium (Figure 1B). We designed a convoluted geometry of microchannels, where the upper (i.e., luminal) and the lower (i.e., vascular) microchannels were fabricated at 500 and 200 µm in height, respectively, separated by a 20-µm-thin flexible porous membrane (Figure 1C), emulating the similar porosity of the human intestinal basement membrane [10].

We performed a computational simulation to characterize the non-linear flow patterns applied in the PMI Chip (Figure 1D). The averaged linear flow velocities in the upper and lower microchannels were approximately 39.7 and 83.0 µm/s, respectively, when the volumetric flow rate was set to 50 µL/h. The 3D hydrodynamics of the flow rate across the upper and the lower microchannels revealed that the directions of the fluid flow lay along with the channel shape (Figure 1D, white arrows in each inset), where a higher flow rate was observed near the inner microchannel wall. The simulation results suggested that the linear flow rate near the inner wall of the microchannel was faster than the location near the outer microfluidic wall (Figure 1D, zoom-in insets). We found that the shear stress applied in the microchannels was higher in the area near the inner wall compared to the region near the outer wall (Figure S1A). In addition, fluid shear stress was significantly higher near the wall than the center of the lower microchannel (Figure S1B). When the cross-sectional shear stress was visualized (Figure S1B), the top ceiling of the lower microchannel showed the highest shear stress, while flow velocity showed an inverse association (Figure S1C). Furthermore, the convoluted PMI Chip has an extended flowing path in the microchannel compared to the original gut-on-a-chip (Figure S2A), by which the fluid residence time is substantially enhanced (approximately 1.8 folds). For instance, when a 50 µL/h volumetric flow rate was applied, which is equivalent to 0.02 dyne/cm^2^ in the upper microchannel, the convoluted PMI Chip has approximately 10.7 min of the residence time, whereas the original gut-on-a-chip shows approximately 6 min of the fluid residence time (Figure S2B).

### 3.2. Multiaxial Stretching Motion in Microchannels

The vacuum chambers next to the convoluted cell microchannels contribute to exert intestinal peristalsis-like deformations, where we anticipated that the PMI Chip undergoes multiaxial stretching motions, whereas the previous linear MPS models do not [10,17]. The convoluted microchannels not only provides the tortuosity of hydrodynamic flow (Figure 1D) but also induces the multiaxial distortions of a flexible porous membrane in response to the cyclic negative pressurization in the vacuum chambers.

**Figure 2:**
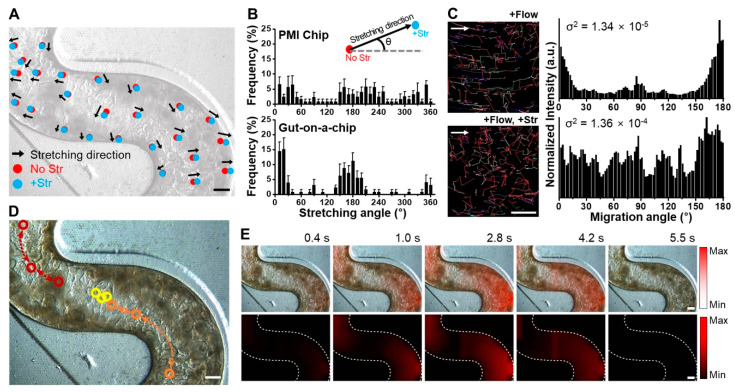
The visualization of multiaxial stretching motions in a PMI Chip. (**A**) A phase contrast micrograph of the normal organoid epithelium cultured in a PMI Chip for 8 days with rhythmical mechanical motions. Bar, 250 μm. The dynamic displacement of multiple locations in the microchannel was tracked without (0% strain, No Str) or with stretching motions (5% cell strain, +Str), where the arrows indicate the direction of stretching. The magnitude of elongation was reflected in the corresponding length of each arrow. The image was adjusted in gray to visualize the multiaxial stretching motions marked by dots and arrows. (**B**) The quantitative profiling of stretching directions as a function of orientation angle in a PMI Chip and a gut-on-a-chip. The PMI Chip shows omnidirectional uniform deformations compared to the gut-on-a-chip. (**C**) The trajectory of fluorescent beads (1 μm in diameter) introduced in the upper microchannel of a PMI Chip under the microfluidic flow alone (+Flow) or the presence of both flow and mechanical deformations (+Flow, +Str). Bar, 100 μm. The corresponding normalized histograms (right panel) quantitatively display the directional movement of randomly selected fluorescent beads (more than 65 particles) in the microchannel of a PMI Chip (σ^2^, variance). (**D**) The tracking of three floating cells (red, yellow, and orange) in response to multiaxial deformations in a cycle of stretching motion in a PMI Chip. Bar, 250 μm. Open circles and dashed arrows indicate the location and trajectory of a target drifting particle, respectively. (**E**) The consecutive time-lapse images of the epithelial layer overlaid with the elongation map that visually highlights a cycle of elongation as a function of color density in a PMI Chip (upper) and a separated channel that independently shows the real-time quantification of the cell elongation in red (lower). The color bars indicate the minimum and maximum intensity of the elongation. Bars, 250 μm.

We quantified the profile of stretching motions at various locations of the microchannels under mechanical deformations. After normal colon organoids (colonoids) formed 3D morphology in the PMI Chip after 10 days of culture, a rhythmical mechanical motion that mimics intestinal bowel movement was applied with approximately 5% in cell strain at 0.15 Hz in frequency. When multiple locations in the microchannel were tracked for quantifying the dynamic displacement between the conditions without (Figure 2A, “No Str”) or with the stretching motions (Figure 2A, “+Str”), the stretching direction of randomly chosen points shows multiaxial displacement with various stretching magnitudes (Figure 2A and Figure S3). The quantitative profiling of stretching directions as a function of angle (Figure 2B, Figures S3 and S4) revealed that the PMI Chip can provide an omnidirectional deformation pattern across the entire convoluted microchannel. On the contrary, the gut-on-a-chip that contains a linear microchannel shows that a perpendicular vacuum stretching resulted in a biased deformation predominantly at around 0°, 180°, and 360°, suggesting a biaxial deformation pattern (Figure 2B). To verify the effect of dynamic stretching motion on the flow pattern, we visualized fluid flow by introducing fluorescent beads in the upper microchannel and analyzed the trajectory of fluorescent beads (Figure 2C, Videos S1 and S2). When the flow alone was applied without multiaxial stretching motions, the migration of fluorescent beads in the upper microchannel was mostly identical to the direction of fluid flow with migrating angles of 0° and 180° (Figure 2C, Video S1). On the contrary, in the presence of both fluid flow and stretching motions, the migrating direction of fluorescent beads was omnidirectional (i.e., a random trajectory without a patterned direction), resulting in the well-distributed migrating angles (Figure 2C, Video S2). Interestingly, we observed that the dynamic stretching motion alone (i.e., without fluid flow) can induce particle migration in the luminal fluid (Figure 2D and Figure S5), suggesting that the integrative biomechanical functions synergize the mixing property, propulsion of particles, and additional fluid shear stress on top of the flow-induced shear stress. Furthermore, the stretching dynamics in a PMI Chip were analyzed by quantifying the cell elongation in real time (Figure 2E, Videos S3 and S4). Within one cycle of the sinusoidal stretching motion (approximately 6.7 s per cycle), the cell elongation initiated from the curvature area, near the vacuum chambers, reached the maximum cell elongation (2.8 s); then afterwards, a gradient of the cell elongation was generated. Notably, the straight-line area also demonstrated that the cell elongation before (2.8 s) and after (4.2 s) the stretching motions reaches to the maximum amplitude (approximately 3.3 s). The stretching dynamics applied to various locations in the convoluted microchannel were distinct as a function of location (Figure S6). However, there was no significant difference in elongation intensity at outer, middle, and inner locations perpendicular to the flow direction (Figure S6).

### 3.3. The 3D Morphogenesis of Intestinal Organoid-Derived Epithelium

**Figure 3:**
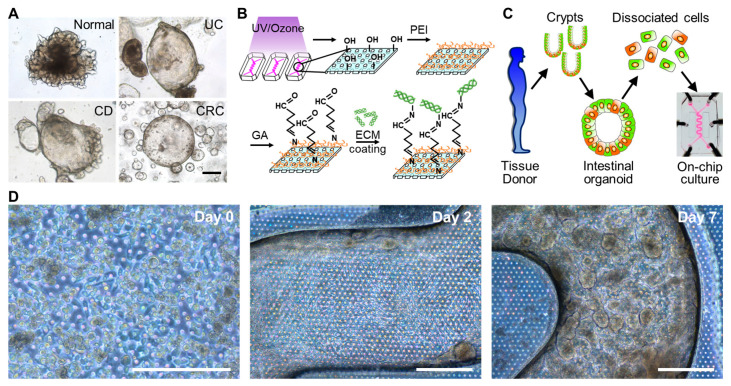
The microfluidic culture of the patient-derived organoid epithelium in a PMI Chip. (**A**) The phase contrast micrographs of human intestinal organoids derived from a normal donor or the patients diagnosed with UC, CD, and CRC. (**B**) The experimental procedure for the activation of a microfluidic channel and a subsequent ECM coating in a PMI Chip. (**C**) The procedure of the formation of biopsy-derived intestinal organoids and its use for a microfluidic culture in a PMI Chip. (**D**) The phase contrast images that show the 3D regeneration of patient-derived intestinal organoid epithelium under non-linear luminal flow and multiaxial stretching motions. Normal organoid cells were cultured in a PMI Chip for 7 days under flow (50 µL/h) and stretching motions (5%, 0.15 Hz). Bars, 200 μm.

To assess the growth of intestinal epithelium on the convoluted PMI Chip, we used intestinal organoids obtained from normal donors or the patients diagnosed with UC, CD, or CRC (Figure 3A and Figure S7). We pretreated the empty microchannel with PEI and GA to modify the hydrophobic PDMS surface, which promotes the efficiency of the ECM coating (Figure 3B) and cell attachment [35]. When the microchannel was saturated with the organoid culture medium, each organoid line pre-cultured in a well plate (Figure S7) was enzymatically disassociated and then seeded in the ECM-coated upper microchannel for the microfluidic on-chip culture (Figure 3C). The constant flow (50 μL/h) and mechanical deformation (5%, 0.15 Hz) applied to the colonoid epithelial monolayer allowed the cells to regenerate a protruded epithelial microarchitecture in 7–10 days depending on the lines from different patients (Figure 3D).

**Figure 4:**
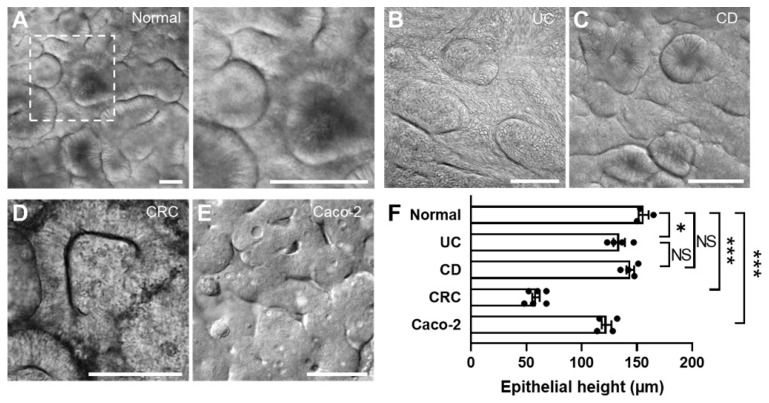
The regeneration of 3D epithelial morphology in a PMI Chip. The DIC micrographs reveal the 3D morphogenesis when the intestinal organoids obtained from normal (**A**) or various patient donors with UC (**B**), CD (**C**), CRC (**D**) as well as a Caco-2 epithelium (**E**) are cultured in a PMI Chip for approximately 150 h under dynamic flow (50 µL/h) and motions (5%, 0.15 Hz). A right inset in “A” is a zoomed-in view of the area indicated by a white dashed box. Bars, 100 μm. (**F**) The height of organoid-derived or Caco-2 intestinal epithelium cultured in a PMI Chip after 150 h. * *p* < 0.05, *** *p* < 0.001. NS, not significant.

The convoluted channel structure did not compromise the uniform cell seeding across the channel (Figure S8). Upon the consistent cell seeding in a PMI Chip, dissociated intestinal organoids reproducibly and robustly regenerated 3D epithelial microarchitectures across multiple different lines and diseases of colonoids (Figure 4). While the organoids derived from a normal donor (Figure 4A and Figure S9A) and patients with UC (Figure 4B) and CD (Figure 4C and Figure S9B) formed similar epithelial structures in the PMI Chip device, CRC organoid cultured in a PMI Chip showed somewhat distinct morphology compared to other lines (Figure 4D). The Caco-2 cells cultured in a PMI Chip also showed a similar 3D epithelial structure (Figure 4E and Figure S9C). The epithelial height profile showed that the height of the CRC epithelium was significantly lower (58.7 ± 8.3 µm) than those of normal epithelium (156.0 ± 7.9 µm) (Figure 4F). Meanwhile, UC (133.9 ± 10.3 µm) and CD (144.0 ± 7.0 µm) epithelial layers did not show a significant difference in height. The epithelium derived from Caco-2 cells showed the lower epithelial height (122.5 ± 8.9 µm) compared to that derived from normal organoids.

**Figure 5:**
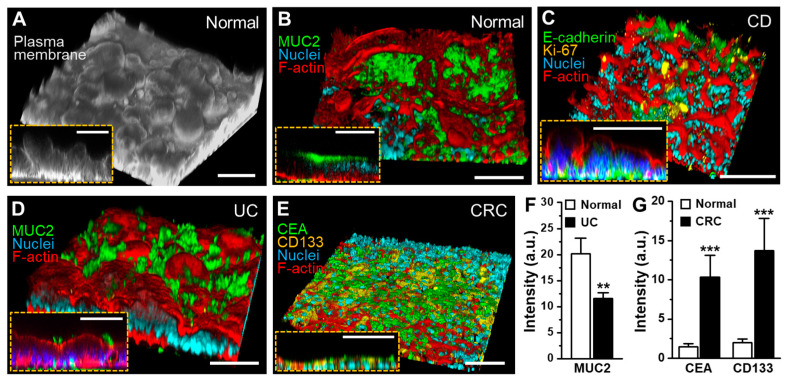
The characterization of the 3D morphology and differentiation of patient-derived organoid epithelium grown in a PMI Chip. The angled views of the 3D epithelial layer derived from (**A**,**B**) normal, (**C**) CD, (**D**) UC, and (**E**) CRC organoids. The insets in the figures from A to E displays the vertical cross-cut view of each 3D epithelial layer. Various structural (F-actin, E-cadherin), functional (MUC2, Ki-67), or disease-specific markers (CEA, CD133) were highlighted by immunofluorescence staining followed by confocal microscopy. As a counterstaining, nuclei were highlighted in cyan. Bars, 100 μm. The quantitative comparisons of the expression of functional (MUC2) (**F**) and disease-specific markers (CEA, CD133) (**G**) in the normal and the patient-derived organoid epithelium in a PMI Chip. ** *p* < 0.01, *** *p* < 0.001.

The regenerated 3D epithelial layers displayed the markers specific to epithelial functions (e.g., MUC2, E-cadherin, Ki-67) or disease characteristics (e.g., CEA, CD133) (Figure 5). The epithelial layers originated from normal (Figure 5A,B), CD (Figure 5C), and UC organoids (Figure 5D) showed the 3D morphology and the spatially localized expression of intestinal structural markers (e.g., F-actin).

The expression of MUC2 was observed near the apical membrane of the established epithelial layer (Figure 5B), whereas the proliferative cells highlighted with Ki-67 were predominantly localized in the lower layer of the CD epithelium (Figure 5C), closely recapitulating the in vivo proliferating signals localized in the basal crypt area. In addition, an adherens junctional protein, E-cadherin, was localized between the cells, indicating that an intact epithelial cell–cell junction was established (Figure 5C, an inset). On the other hand, MUC2 expression on the UC epithelium was found to be significantly lower than that on the normal epithelium (Figure 5D,F), which shows a good agreement with the decreased MUC2 expression in UC patients [36,37]. Interestingly, the CRC epithelium did not show 3D morphogenesis compared to other groups (Figure 5E). The expression of CRC markers such as CEA and CD133 was observed throughout the CRC epithelial layer grown in a PMI Chip. It is noted that the normal organoid-derived epithelium minimally expressed the CEA and CD133, suggesting that the non-tumor characteristics were confirmed in the normal control (Figure 5G and Figure S10).

### 3.4. Co-Culture of the Human Fecal Microbiome in a PMI Chip

**Figure 6:**
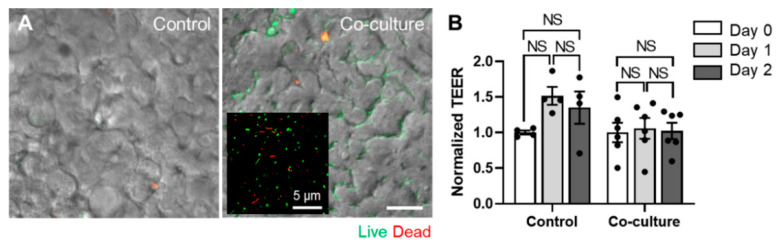
The co-culture of the fecal microbiome in a PMI Chip lined by 3D intestinal epithelium under an AOI. (**A**) The overlaid images of a DIC and a fluorescent micrograph show the snapshot of a host–microbiome co-culture in the PMI Chip. The human fecal microbiome (initial seeding density, 5 × 10^4^ cells/mL) was co-cultured with the germ-free Caco-2 intestinal epithelium in a PMI Chip under luminal flow (100 µL/h) and multiaxial stretching motions (5% in cell strain, 0.15 Hz in frequency) for 2 days. The AOI was pre-established by perfusing an antibiotic-free anoxic and oxic culture medium in the upper and lower microchannel, respectively for 12 h. The fecal bacteria that formed microcolonies were visualized using Live/Dead staining dye in the lumen microchannel. We performed the same staining into the germ-free PMI Chip (Control). An inset in the “Co-culture” shows the viability of the isolated human fecal microbiome acquired by confocal microscopy. (**B**) The epithelial barrier function was maintained (> 3 kΩ·cm^2^) regardless of the AOI or the co-culture with the fecal microbiome. The barrier function was assessed by normalizing TEER values. Bar, 50 µm. NS, not significant.

To demonstrate the patient’s mucosal microenvironment and dynamic host–microbiome crosstalk at various GI diseases, the co-culture of the patient-derived gut microbiome on the patient-derived mucosal interface is essential. As a proof of principle, we obtained a healthy donor’s fecal microbiome and demonstrated a stable co-culture with the established Caco-2 epithelium in the convoluted PMI Chip. We confirmed that the fecal microbiome applied in the AOI-pre-established PMI Chip shows stable growth with high viability (> 80% viable populations) over multiple days of co-cultures (Figure 6A). We also verified that the co-culture with the fecal microbiome does not compromise the epithelial barrier function quantitated by TEER over time up to 48 h (Figure 6B). In addition, after 48 h of the co-culture, bacterial colonies were formed between the epithelial folds (Figure 6A, “Co-culture”).

## 4. Discussion

In this study, we report a PMI Chip that contains an advanced design with the non-linear hydrodynamic flow and multiaxial cellular deformations, mimicking the physiodynamic and mechanical cues in the intestinal microenvironment. In the PMI Chip, intestinal epithelial cells derived from a normal donor or the patients with various GI diseases reproducibly generated the 3D epithelial layer. In addition, the PMI Chip enabled co-culture of the human fecal microbiome with the established 3D epithelium to recapitulate patient-specific host–microbiome crosstalk germane to the disease development.

We substantially improved the original design of the gut-on-a-chip that possesses a linear cell microchannel [10,11,12,13,14,15,16,30] into the convoluted 3D microchannels that generate the multiaxial cell strains and physiologically relevant flow regime. Our new design principle that emulates the non-linear flow pattern dramatically induced multiaxial deformations in response to the bidirectional vacuum-driven elongations, where no complex mechanical manifold valves or controlling module is necessary to induce the complex mechanical dynamics of the living human gut. In addition, the increased culture area and the residence time of microfluidic flow in a convoluted PMI Chip allow the intestinal epithelium to sufficiently interact with the surrounding environment, which can elicit active intercellular responses. The enhanced residence time in a PMI Chip may also be beneficial to perform an accurate transport assay for assessing the absorption or efflux of various drug candidates [38,39].

The multiaxial deformations in the PMI Chip were validated by (1) visualizing and analyzing the profile of the stretching motions (direction and magnitude) at various locations of microchannels in a PMI Chip, (2) quantitative assessment of the stretching direction as a function of orientation angle, and (3) particle mixing in the presence of the stretching motion. In addition, we confirmed that the cells bound on a porous membrane undergo a corresponding cell strain and physical movement in response to the multiaxial deformations that the membrane exerts. The integrative biomechanical functions of dynamic fluid flow and multiaxial stretching deformations synergize to promote the mixing property, propulsion of particles, and additional fluid shear stress on top of the flow-induced shear stress. This peristalsis-like deformation is germane to the complex bowel movement that includes macroscopic mixing (e.g., segmental) and propulsion as well as microscopic villus motility (e.g., swinging, sliding, and piston-like vibrations) [40]. Thus, the multiaxial deformation may contribute to emulate the complex biomechanical component.

The luminal velocity and shear stress in the PMI Chip are physiologically comparable to the ones found in the human intestine [41,42,43,44,45]. The convoluted curvature of microchannels leads to asymmetric flow profile, which is similar to the flow pattern found in the gut [46,47]. The apical and basolateral regions of the intestinal mucosal interface experience different flow velocity, where the flow rate in the submucosal capillary vasculature is higher than that in the lumen surface. From this physiological perspective, the differences in luminal velocity and shear stress between upper and lower microchannels can elicit the physiological hydrodynamics of the intestinal mucosal interface. On the other hand, we also calculated a pressure difference between the upper and lower microchannels, where approximately 0.59 Pa of the pressure difference can be induced between the upper and lower microchannels. This pressure difference resulted in approximately 0.009 cm/s of flow through the membrane holes. However, this flow did not compromise the cell attachment in the experimental setup.

The intestinal organoid culture has been carried out in a static condition, which remains a potential limitation because the mechanically dynamic culture condition can potentially orchestrate the phenotype of the cultured cells in vitro [10,11,26]. Furthermore, the enclosed lumen in an organoid body considerably hampers access to the lumen, by which the co-culture with gut microbiome or the simulation of drug administration is extremely challenging. Thus, this limitation led to the innovation of an “opened” version of an organoid, where the recreation of a mucosal tissue interface of intestinal organoids has been emerged [6,48,49]. On top of the previous reports [20,50], we propose an advanced 3D convoluted microdevice that can manipulate multiaxial deformations and independently controlled fluid hydrodynamics in the mucosal interface. Moreover, our study successfully reveals that the donor-specific organoid-based microfluidic cultures can potentially claim the patient surrogate that reflects genetic and pathophysiological characteristics in various GI diseases including IBD and CRC. Our approach has a couple of advanced features. First, it provides an accessible tissue interface in both luminal (i.e., apical) and vascular compartment (i.e., basolateral), by which the directional introduction of necessary components is possible. The PMI Chip can remarkably improve the limitation of access to the enclosed lumen in the 3D organoids. Second, mechanically defined deformations and fluid hydrodynamics can be applied to the organoid-derived epithelium, which is extremely challenging, or even impossible, in the 3D organoid cultures. Third, stable co-culture with a host microbiome can be controllable for a longitudinal study. In particular, the creation of AOI enables growing and maintaining obligate an anaerobic gut microbiota that induces critical host–microbiome crosstalk pertinent to the disease etiology and development. Finally, real-time monitoring of the cell morphology and cell–cell interactions can be possible by adapting multiple imaging modalities. As previously proved [10,15,16], co-cultured epithelial and microbial cells can be visualized with a cellular and molecular resolution by applying phase contrast, DIC, immunofluorescence, or microfluorimetry microscopy in a timely manner. All these advances will contribute to the building of a patient-specific model to explore clinical and pharmacological questions.

## 5. Conclusion

We report the PMI Chip that has a convoluted microchannel and a multiaxial deformable module. The verification of the utility of the PMI Chip by adapting the patient-derived organoid epithelium confirms the potential applicability, accessibility, and modularity of the PMI Chip. We envision that the implementable and disseminating features of the PMI Chip should be further evaluated by collaborating GI clinicians and oncologists, immunologists, and microbiologists for faithfully contributing to the Precision Medicine.

## Supplementary Materials

The following are available online at https://www.mdpi.com/2072-666X/11/7/663/s1, Figure S1: Computational simulation of the fluid dynamics in a PMI Chip. (A) Profiles of fluid shear stress in the upper and lower microchannels of the PMI Chip at 50 μL/h. Cross-sectional visualization of (B) shear stress and (C) flow velocity at the location designated with white dotted lines in (A). Figure S2: The profile of fluid residence time in a PMI Chip and a gut-on-a-chip. (A) Comparisons of flowing path between the convoluted PMI Chip and a gut-on-a-chip. (B) The estimated residence time in each chip at 30, 50, and 100 μL/h, respectively. Figure S3: Multiaxial stretching motions at multiple locations in a microchannel. (A) A schematic of a PMI Chip displays the structure of the convoluted design of microchannels, where a colored area indicates the region of co-culture. (B) Phase contrast micrographs of the normal organoid-derived epithelium cultured in the PMI Chip for 8 days, before (left; 0% strain) and after stretching motions (right; 5% cell strain). (C) Multiaxial stretching motions at the left curved corner position of the PMI Chip. Red and light blue dots were randomly set out to trace the elongated positions. Bar, 200 μm. Figure S4: Monoaxial deformation patterns of multiple locations in a gut-on-a-chip. (A) A schematic of a gut-on-a-chip, where a colored area indicates the region of co-culture. (B) Monoaxial stretching motions in the linear microchannel of a gut-on-a-chip without (0% strain; red dots) or with the stretching motions (5% cell strain; light blue dots). A phase contrast image was divided into 16 squared compartments of equal area (black dotted lines), and two dots were randomly positioned in each compartment to track the directionality of stretching. Bar, 200 μm. Figure S5: Consecutive time-lapse images of particle movements induced by multiaxial stretching motions in a PMI Chip. A floating cell was tracked by checking the individual frame in a recorded video file. Normal organoid cells were cultured for 8 days in the PMI Chip. Bar, 200 μm. Figure S6: Multiaxial stretching dynamics applied to various locations in the convoluted microchannels of a PMI Chip. Phase contrast micrographs of the human organoid-derived epithelium grown in the left (A) or right corner (B) of a PMI Chip were analyzed before (No strain) and after stretching motions (+Strain). The % elongation at each position (1, 2, and 3) designated on the white dashed line is quantified in the column charts. Quantitative assessment of the stretching intensity monitored at the left (C) and right corner (D) of a cell-free convoluted PMI Chip. Quantification of % elongation is provided in the column charts next to each phase contrast snapshot. Normal organoid cells were cultured for 8 days in the PMI Chip. Bars, 200 μm. NS, not significant. Figure S7: Morphological observations for the in vitro growth of human intestinal organoids. Phase contrast micrographs of organoids derived from (A) normal donors or patients diagnosed with (B) UC, (C) CD, or (D) CRC. Phase contrast micrographs were acquired at a fixed position on Day 1, 3, and 7 upon the passage. Figure S8: A uniform seeding of the dissociated organoid epithelium in a PMI Chip. Phase contrast images taken at the inlet (A), right corner (B), and left corner (C) reveal the uniform distribution of dissociated organoid cells after seeding. Cell density was adjusted at ~1×10^7^ cells/mL. The lower panel of the image is a zoom-in view of a white dashed box shown in the upper panel. Bars, 500 µm. Figure S9: Morphological observations for the 3D intestinal epithelium in a PMI Chip. Intestinal epithelium derived from (A) normal organoids cultured for 10 days, (B) CD organoids (CD7517) cultured for 8 days, and (C) Caco-2 cells cultured for 7 days in the PMI Chips, respectively. Bars, 500 μm. Figure S10: Characterization of the 3D morphology and the expression of disease-specific markers (CEA, CD133) of the normal organoid epithelium grown in a PMI Chip. Structural (F-actin) and disease-specific markers (CEA, CD133) were immunofluorescence stained, followed by laser-scanning confocal microscopy. As a counterstaining, nuclei were highlighted in cyan. An inset displays a vertical crosscut view of the 3D microarchitecture of the normal epithelium. Normal organoids were cultured for 14 days in the PMI Chip. Bars, 100 μm. Video S1: Consecutive time-lapse imaging of fluid flow in the convoluted microchannel of a PMI Chip without stretching motions. Fluid flow was visualized by perfusing fluorescent beads (mean diameter, 1 μm) into the upper microchannel in a PMI Chip at a flow rate of 50 μL/h. Consecutive time-lapse images were acquired with a laser-scanning confocal microscope for 1 min at the time interval of 1 s. The movie was reconstituted from the consecutive time-lapse images with 6 frames/s. Video S2: Consecutive time-lapse imaging of fluid flow in the convoluted microchannel of a PMI Chip with cyclic stretching motions (5%, 0.15 Hz). The migrating direction of fluorescence beads showed an omnidirectional deformation pattern regardless of the angle. Consecutive time-lapse images were acquired with a laser-scanning confocal microscope for 1 min at the time interval of 1 s. The movie was reconstituted from the consecutive time-lapse images with 6 frames/s. Video S3: Real-time visualization for the extension of an epithelial layer in a PMI Chip. The elongation of an epithelial layer was recorded during one cycle of the sinusoidal stretching motion using an inverted phase contrast microscope equipped with a 5× objective (5% in cell strain, 0.15 Hz in frequency). The radial gradient of red dots was overlaid with the recorded video file. To form the epithelial layer, normal organoid cells were cultured in the PMI Chip for 8 days under non-linear flow (50 µL/h) and stretching motions (5%, 0.15 Hz). Video S4: Color-coded mapping for the elongation degree of the microchannel in a PMI Chip. The color coding was performed by analyzing an array of motion tracking points designated on the target area in the movie frame.
