# Supplementary material for: Three-Dimensional Regeneration of Patient-Derived Intestinal Organoid Epithelium in a Physiodynamic Mucosal Interface-on-a-Chip
Source: Micromachines (Basel). 2020 Jul 7;11(7):663. doi: 10.3390/mi11070663 (PMC7408321; doi:10.3390/mi11070663)
Supplement: Supplementary file 1 [file micromachines-11-00663-s001.zip › micromachines-806101-proofed-si/Supplementary Materials.SUBMIT.pdf]

micromachines

Supplementary Materials: Three-Dimensional Regeneration of Patient-Derived Intestinal Organoid Epithelium in a Physiodynamic Mucosal Interface-on-a-Chip

Yong Cheol Shin, Woojung Shin, Domin Koh, Alexander Wu, Yoko M. Ambrosini, Soyoun Min, S. Gail Eckhardt, R. Y. Declan Fleming, Seung Kim, Sowon Park, Hong Koh, Tae Kyung Yoo and Hyun Jung Kim

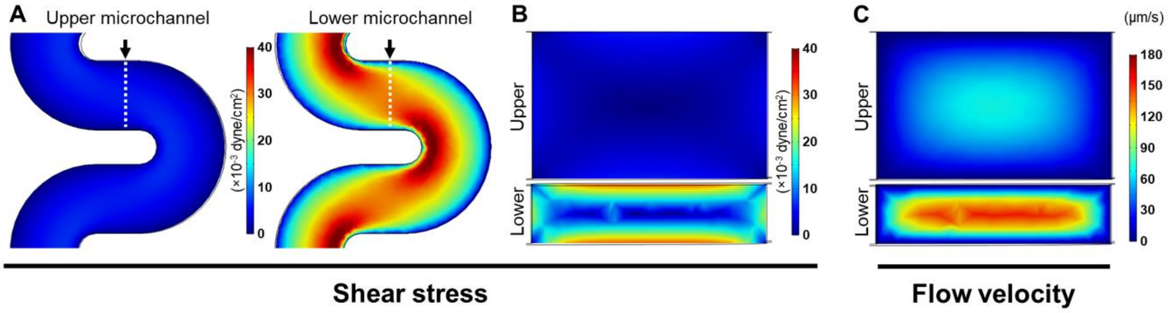

Figure S1. Computational simulation of the fluid dynamics in a PMI Chip. **(A)** Profiles of fluid shear stress in the upper and lower microchannels of the PMI Chip at 50 µL/h. Cross-sectional visualization of **(B)** shear stress and **(C)** flow velocity at the location designated with white dotted lines in (A).

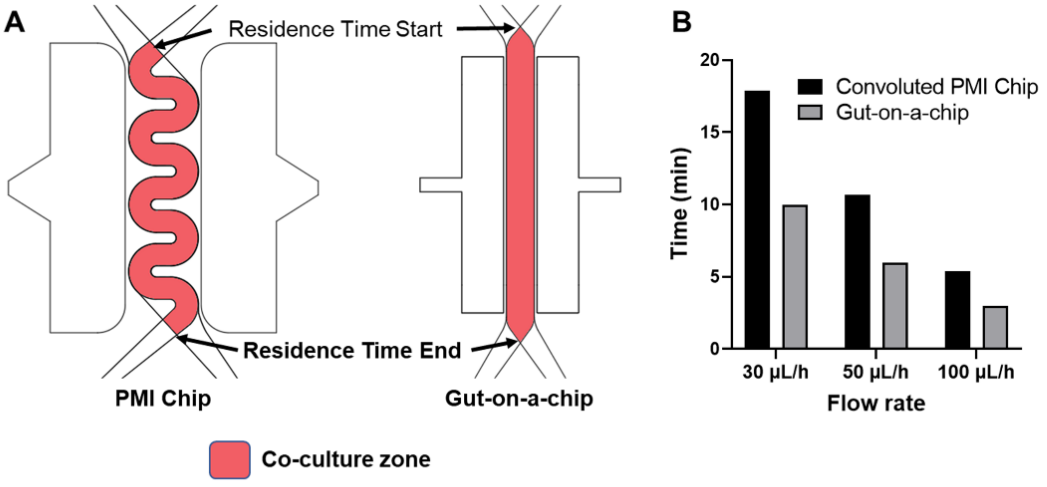

Figure S2. The profile of fluid residence time in a PMI Chip and a gut-on-a-chip. **(A)** Comparisons of flowing path between the convoluted PMI Chip and a gut-on-a-chip. **(B)** The estimated residence time in each chip at 30, 50, and 100 µL/h, respectively.

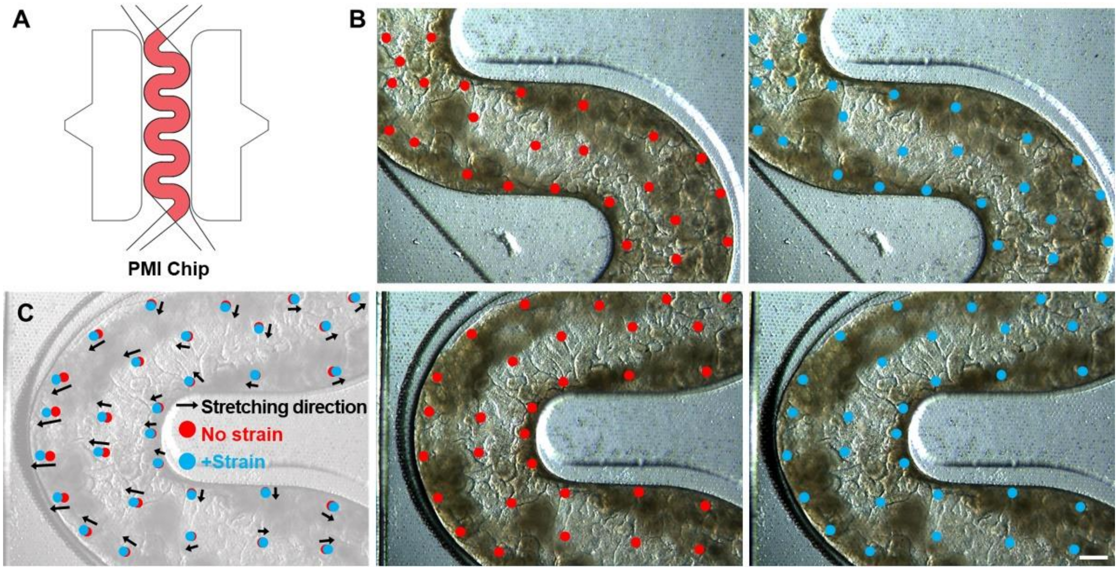

Figure S3. Multiaxial stretching motions at multiple locations in a microchannel. **(A)** A schematic of a PMI Chip displays the structure of the convoluted design of microchannels, where a colored area indicates the region of co-culture. **(B)** Phase contrast micrographs of the normal organoid-derived epithelium cultured in the PMI Chip for 8 days, before (left; 0% strain) and after stretching motions (right; 5% cell strain). **(C)** Multiaxial stretching motions at the left curved corner position of the PMI Chip. Red and light blue dots were randomly set out to trace the elongated positions. Bar, 200 µm.

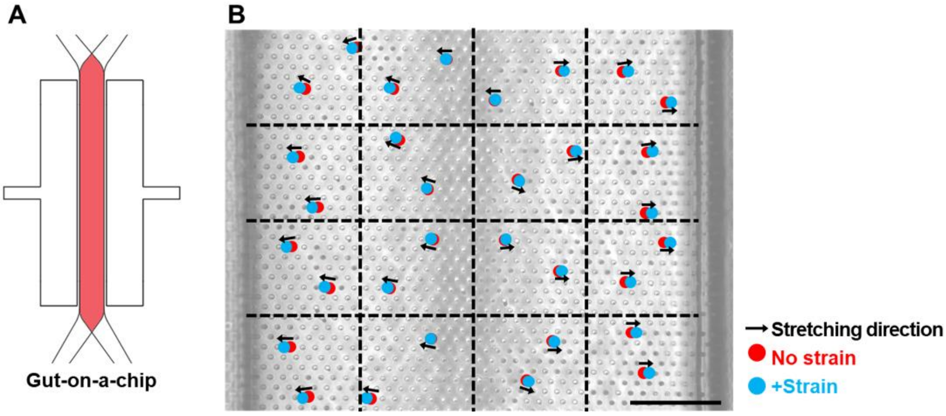

Figure S4. Monoaxial deformation patterns of multiple locations in a gut-on-a-chip. **(A)** A schematic of a gut-on-a-chip, where a colored area indicates the region of co-culture. **(B)** Monoaxial stretching motions in the linear microchannel of a gut-on-a-chip without (0% strain; red dots) or with the stretching motions (5% cell strain; light blue dots). A phase contrast image was divided into 16 squared compartments of equal area (black dotted lines), and two dots were randomly positioned in each compartment to track the directionality of stretching. Bar, 200 µm.

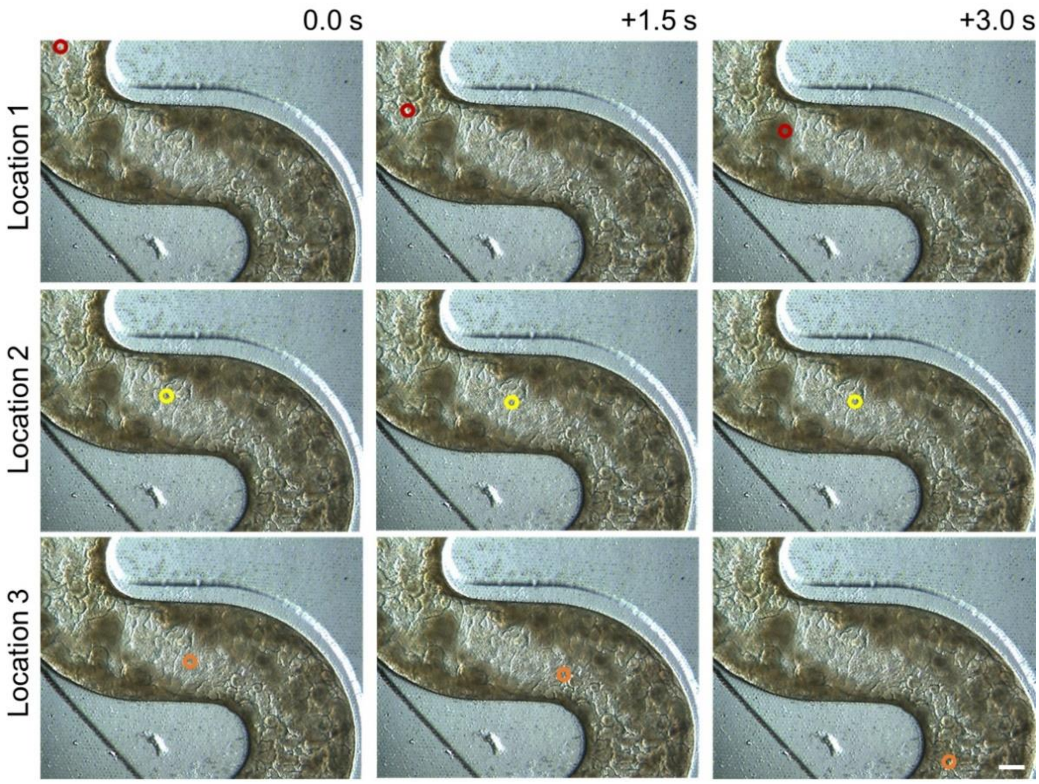

Figure S5. Consecutive time-lapse images of particle movements induced by multiaxial stretching motions in a PMI Chip. A floating cell was tracked by checking the individual frame in a recorded video file. Normal organoid cells were cultured for 8 days in the PMI Chip. Bar, 200 μm.

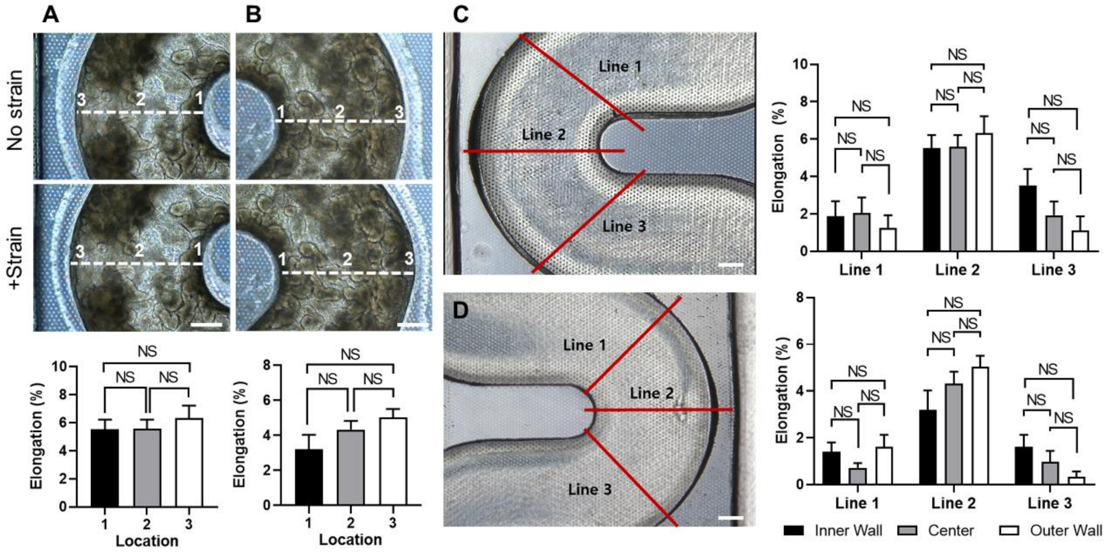

Figure S6. Multiaxial stretching dynamics applied to various locations in the convoluted microchannels of a PMI Chip. Phase contrast micrographs of the human organoid-derived epithelium grown in the left **(A)** or right corner **(B)** of a PMI Chip were analyzed before (No strain) and after stretching motions (+Strain). The % elongation at each position (1, 2, and 3) designated on the white dashed line is quantified in the column charts. Quantitative assessment of the stretching intensity monitored at the left **(C)** and right corner **(D)** of a cell-free convoluted PMI Chip. Quantification of % elongation is provided in the column charts next to each phase contrast snapshot. Normal organoid cells were cultured for 8 days in the PMI Chip. Bars, 200 μm. NS, not significant.

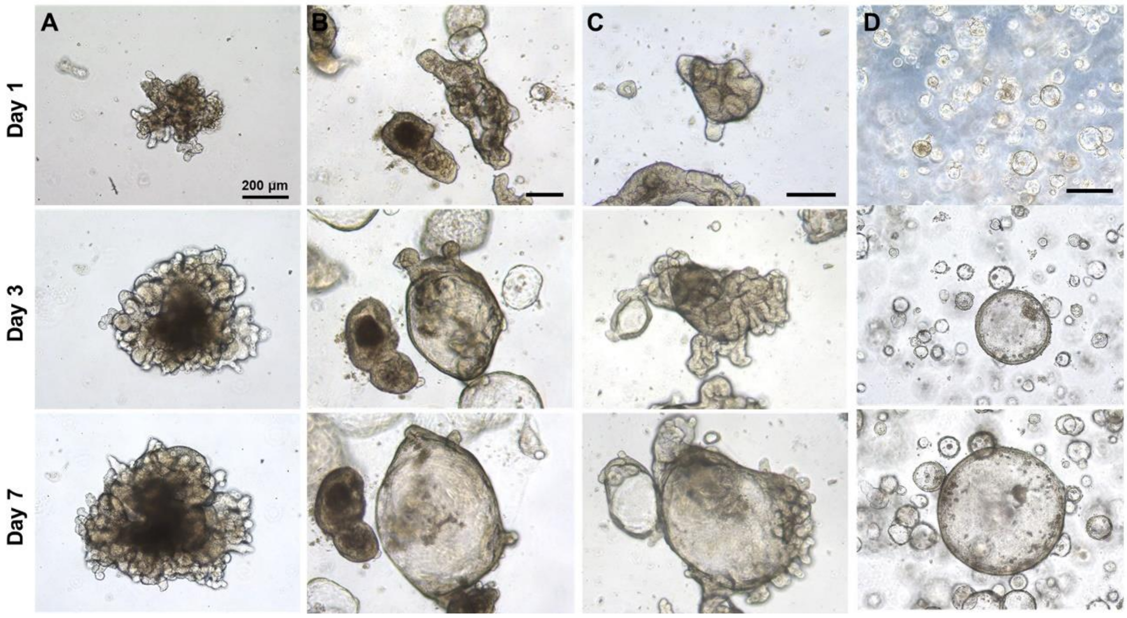

Figure S7. Morphological observations for the *in vitro* growth of human intestinal organoids. Phase contrast micrographs of organoids derived from **(A)** normal donors or patients diagnosed with **(B)** UC, **(C)** CD, or **(D)** CRC. Phase contrast micrographs were acquired at a fixed position on Day 1, 3, and 7 upon the passage.

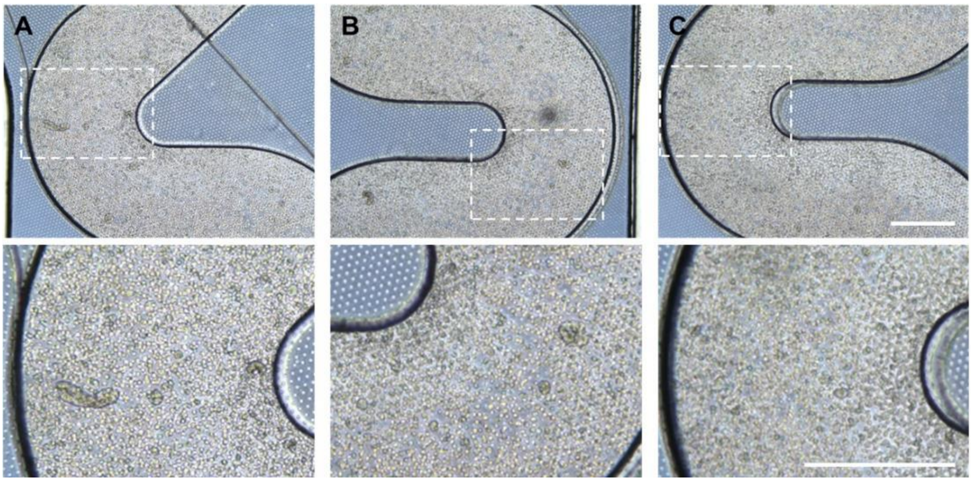

Figure S8. A uniform seeding of the dissociated organoid epithelium in a PMI Chip. Phase contrast images taken at the inlet **(A)**, right corner **(B)**, and left corner **(C)** reveal the uniform distribution of dissociated organoid cells after seeding. Cell density was adjusted at ~1×10^7 cells/mL. The lower panel of the image is a zoom-in view of a white dashed box shown in the upper panel. Bars, 500 μm.

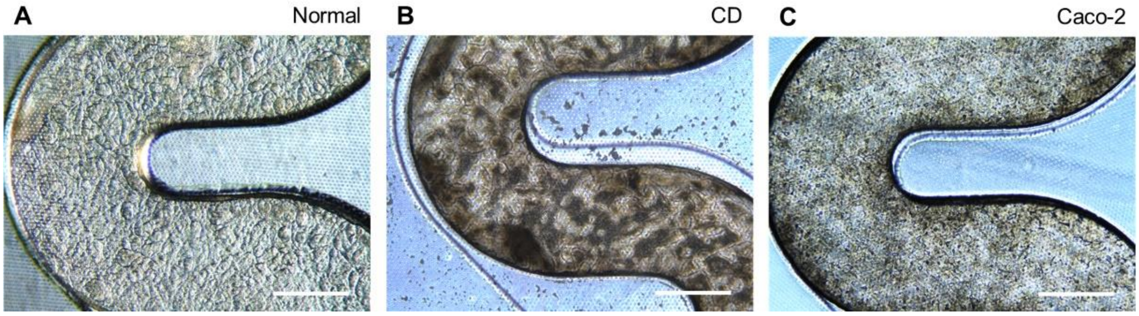

Figure S9. Morphological observations for the 3D intestinal epithelium in a PMI Chip. Intestinal epithelium derived from **(A)** normal organoids cultured for 10 days, **(B)** CD organoids (CD7517) cultured for 8 days, and **(C)** Caco-2 cells cultured for 7 days in the PMI Chips, respectively. Bars, 500 μm.

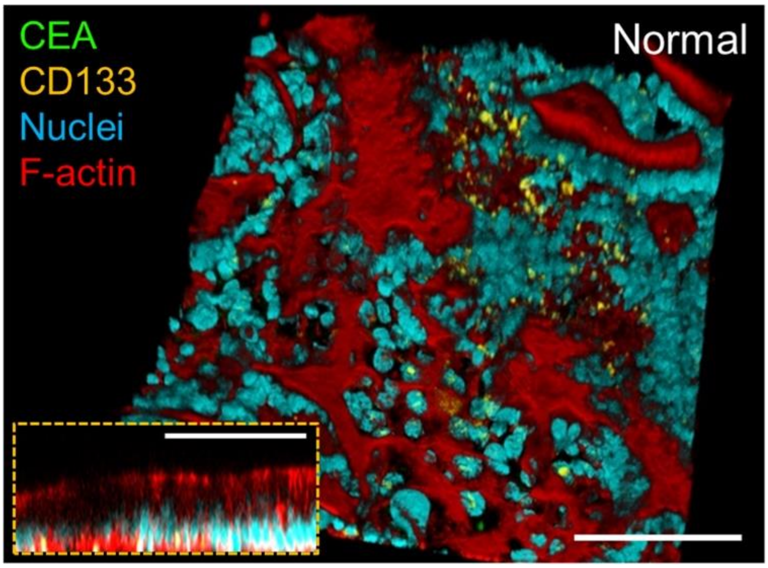

Figure S10. Characterization of the 3D morphology and the expression of disease-specific markers (CEA, CD133) of the normal organoid epithelium grown in a PMI Chip. Structural (F-actin) and disease-specific markers (CEA, CD133) were immunofluorescence stained, followed by laser-scanning confocal microscopy. As a counterstaining, nuclei were highlighted in cyan. An inset displays a vertical crosscut view of the 3D microarchitecture of the normal epithelium. Normal organoids were cultured for 14 days in the PMI Chip. Bars, 100 μm.

Legends for Supplementary Videos

Supplementary Video S1. Consecutive time-lapse imaging of fluid flow in the convoluted microchannel of a PMI Chip without stretching motions. Fluid flow was visualized by perfusing fluorescent beads (mean diameter, 1 μm) into the upper microchannel in a PMI Chip at a flow rate of 50 μL/h. Consecutive time-lapse images were acquired with a laser-scanning confocal microscope for 1 min at the time interval of 1 s. The movie was reconstituted from the consecutive time-lapse images with 6 frames/s.

Supplementary Video S2. Consecutive time-lapse imaging of fluid flow in the convoluted microchannel of a PMI Chip with cyclic stretching motions (5%, 0.15 Hz). The migrating direction of fluorescence beads showed an omnidirectional deformation pattern regardless of the angle. Consecutive time-lapse images were acquired with a laser-scanning confocal microscope for 1 min at the time interval of 1 s. The movie was reconstituted from the consecutive time-lapse images with 6 frames/s.

Supplementary Video S3. Real-time visualization for the extension of an epithelial layer in a PMI Chip. The elongation of an epithelial layer was recorded during one cycle of the sinusoidal stretching motion using an inverted phase contrast microscope equipped with a 5× objective (5% in cell strain, 0.15 Hz in frequency). The radial gradient of red dots was overlaid with the recorded video file. To form the epithelial layer, normal organoid cells were cultured in the PMI Chip for 8 days under non-linear flow (50 μL/h) and stretching motions (5%, 0.15 Hz).

Supplementary Video S4. Color-coded mapping for the elongation degree of the microchannel in a PMI Chip. The color coding was performed by analyzing an array of motion tracking points designated on the target area in the movie frame.
